# Supplementary material for: High-accuracy spinal alignment monitoring using the head angle and visual distance in computer users
Source: PLoS One. 2025 Jun 27;20(6):e0326431. doi: 10.1371/journal.pone.0326431 (PMC12204535; doi:10.1371/journal.pone.0326431)
Supplement: S1 Table — (DOCX) [file pone.0326431.s003.docx]

Supplemental Table 1

Demographic data of the healthy volunteers for 3D motion capture analysis.

|  | Male | Female |
| --- | --- | --- |
| Number of volunteers | 10 | 11 |
| Age (years) | 20-47 (23) | 21-57 (40) |
| Height (cm) | 160-179 (174) | 154-165 (161) |
| Body weight (kg) | 50-89 (64) | 44-65 (51) |
| Body mass index | 16.7-28.4 (21.0) | 17.6-24.8 (19.7) |

3D: three-dimensional, ( ): median
