# Supplementary material for: High-accuracy spinal alignment monitoring using the head angle and visual distance in computer users
Source: PLoS One. 2025 Jun 27;20(6):e0326431. doi: 10.1371/journal.pone.0326431 (PMC12204535; doi:10.1371/journal.pone.0326431)
Supplement: S2 Table — (DOCX) [file pone.0326431.s004.docx]

Supplemental Table 2

Demographic data of the patients for comparative analysis of sagittal spinal alignment between radiographic parameters and angles measured by body surface landmarks.

|  | Male | Female |
| --- | --- | --- |
| Number of patients | 4 | 28 |
| Age (years) | 41-51(47.5) | 37-54 (47) |
| Height (cm) | 168-178 (171) | 140-172 (159) |
| Body weight (kg) | 53-106 (69) | 40-91 (49) |
| Body mass index | 14.1-36.3 (19.3) | 16.6-36.7 (23.9) |

(): median
