# Supplementary material for: High-accuracy spinal alignment monitoring using the head angle and visual distance in computer users
Source: PLoS One. 2025 Jun 27;20(6):e0326431. doi: 10.1371/journal.pone.0326431 (PMC12204535; doi:10.1371/journal.pone.0326431)
Supplement: S3 Table — (DOCX) [file pone.0326431.s005.docx]

Supplemental Table 3

Interrater correlation coefficients (ICC) of X-ray measurements

|  | ICC(1,1) | ICC(2,1) | |
| --- | --- | --- | --- |
|  |  | Ex. 1-1^st^ vs Ex. 2 | Ex. 1-2^nd^ vs Ex. 2 |
| Body surface parameters |  |  |  |
| C2-C7 tilt angle | 0.6946 | 0.8715 | 0.6104 |
| C7-T3 tilt angle | 0.9232 | 0.9046 | 0.8992 |
| C7-T3-T8 angle | 0.7176 | 0.6401 | 0.6303 |
| T3-T8-T12 angle | 0.8703 | 0.8896 | 0.8308 |
| T12-L3-S1 angle | 0.5612 | 0.6662 | 0.5573 |
| X-ray parameters |  |  |  |
| COG-C7 SVA | 0.9413 | 0.9455 | 0.9386 |
| C2-C7 SVA | 0.9721 | 0.9844 | 0.9657 |
| Cervical lordosis | 0.9477 | 0.7168 | 0.6556 |
| T1 slope | 0.9534 | 0.8142 | 0.7489 |
| Upper thoracic kyphosis | 0.9704 | 0.8000 | 0.7756 |
| Lower thoracic kyphosis | 0.8787 | 0.8225 | 0.7474 |
| Lumbar lordosis | 0.8172 | 0.8996 | 0.8905 |

ICC(1,1): intra-observer agreement, ICC(2, 1): inter-observer agreement, Ex.: examiner,

COG: center of gravity, SVA: sagittal vertical axis
