# Supplementary material for: High-accuracy spinal alignment monitoring using the head angle and visual distance in computer users
Source: PLoS One. 2025 Jun 27;20(6):e0326431. doi: 10.1371/journal.pone.0326431 (PMC12204535; doi:10.1371/journal.pone.0326431)
Supplement: S4 Table — (DOCX) [file pone.0326431.s006.docx]

Supplemental Table 4

Coefficients of quadratic linear approximation (estimation formula) for C2-C7 tilt angle

|  | Coefficient | | | | | | | | | | | |  |
| --- | --- | --- | --- | --- | --- | --- | --- | --- | --- | --- | --- | --- | --- |
| Incorporated variable(s) | b_1_ (HA) | b_2_ (HA)^2^ | b_3_ (VD) | b_4_ (VD)^2^ | b_5_ (S) | b_6_ (A) | b_7_ (A)^2^ | b_8_ (H) | b_9_ (H)^2^ | b_10_ (W) | b_11_ (W)^2^ | b_12_ | |
| HA | 0.4849 | 3.114x10^-3^ |  |  |  |  |  |  |  |  |  | 11.10 | |
| VD |  |  | -8.358x10^-2^ | 1.617x10^-5^ |  |  |  |  |  |  |  | 72.87 | |
| HA VD | -9.934x10^-2^ | 7.317x10^-3^ | -5.009x10^-2^ | -5.918x10^-7^ |  |  |  |  |  |  |  | 54.76 | |
| HA VD S | -4.011x10^-2^ | 5.515x10^-3^ | -6.651x10^-2^ | 1.496x10^-5^ | -5.808 |  |  |  |  |  |  | 59.44 | |
| HA VD A | -1.371x10^-2^ | 5.528x10^-3^ | -5.888x10^-2^ | 4.878x10^-6^ |  | 2.253 | -2.723x10^-2^ |  |  |  |  | 16.36 | |
| HA VD H | -9.973x10^-2^ | 7.437x10^-3^ | -4.673x10^-2^ | -2.942x10^-6^ |  |  |  | -8.765 | 2.654x10^-2^ |  |  | 775.7 | |
| HA VD W | -0.1110 | 7.534x10^-3^ | -4.587x10^-2^ | -4.029x10^-6^ |  |  |  |  |  | -0.756 | 6.984x10^-3^ | 73.30 | |
| HA VD S A H W | -4.878x10^-2^ | 4.905x10^-3^ | -6.312x10^-2^ | 9.943x10^-6^ | -3.865 | 2.195 | -2.801x10^-2^ | -11.63 | 3.503x10^-2^ | -6.525x10^-2^ | 6.391x10^-4^ | 985.4 | |
