# Supplementary material for: High-accuracy spinal alignment monitoring using the head angle and visual distance in computer users
Source: PLoS One. 2025 Jun 27;20(6):e0326431. doi: 10.1371/journal.pone.0326431 (PMC12204535; doi:10.1371/journal.pone.0326431)
Supplement: S5 Table — (DOCX) [file pone.0326431.s007.docx]

Supplemental Table 5

Coefficients of quadratic linear approximation (estimation formula) for C7-T3 tilt angle

|  | Coefficient | | | | | | | | | | | |  |
| --- | --- | --- | --- | --- | --- | --- | --- | --- | --- | --- | --- | --- | --- |
| Incorporated variable(s) | b_1_ (HA) | b_2_ (HA)^2^ | b_3_ (VD) | b_4_ (VD)^2^ | b_5_ (S) | b_6_ (A) | b_7_ (A)^2^ | b_8_ (H) | b_9_ (H)^2^ | b_10_ (W) | b_11_ (W)^2^ | b_12_ | |
| HA | 0.5997 | 1.297x10^-3^ |  |  |  |  |  |  |  |  |  | 14.17 | |
| VD |  |  | -0.1542 | 5.665x10^-5^ |  |  |  |  |  |  |  | 104.3 | |
| HA VD | -8.494x10^-2^ | 4.206x10^-3^ | -0.1358 | 4.660x10^-5^ |  |  |  |  |  |  |  | 95.30 | |
| HA VD S | -0.3672 | 7.854x10^-3^ | -0.1026 | 1.512x10^-5^ | 11.76 |  |  |  |  |  |  | 85.82 | |
| HA VD A | -0.1888 | 5.386x10^-3^ | -0.1276 | 3.777x10^-5^ |  | 0.2803 | -7.181x10^-3^ |  |  |  |  | 95.15 | |
| HA VD H | -0.3234 | 6.586x10^-3^ | -0.1147 | 2.411x10^-5^ |  |  |  | 15.01 | -4.324x10^-2^ |  |  | -1.203x10^3^ | |
| HA VD W | -0.2596 | 5.825x10^-3^ | -0.1235 | 3.209x10^-5^ |  |  |  |  |  | 3.410 | -2.439x10^-2^ | -16.26 | |
| HA VD S A H W | -0.4344 | 8.077x10^-3^ | -0.103 | 1.199x10^-5^ | 14.56 | 1.0399 | -1.334x10^-2^ | 6.286 | -1.958x10^-2^ | 2.203 | -1.692x10^-2^ | -502.3 | |
